# Assessment of greenhouse technologies on the drying behavior of cocoa beans

**DOI:** 10.1002/fsn3.1565

**Published:** 2020-04-16

**Authors:** Frederick D. Banboye, Martin N. Ngwabie, Silvia A. Eneighe, Divine B. Nde

**Affiliations:** ^1^ Department of Food and Bioresource Technology College of Technology University of Bamenda Bamenda Cameroon; ^2^ Department of Agricultural and Environmental Engineering College of Technology University of Bamenda Bamenda Cameroon

**Keywords:** cocoa beans, fleece, greenhouse dryer, open sun dryer

## Abstract

Cocoa beans (*Theobroma cacao* L.) are the principal raw material for chocolate manufacture. Before cocoa beans are ready for the chocolate industry, farm‐based fermentation and drying processes are key determinants of bean quality and hence the price. To improve its value, cocoa beans were dried in a modified greenhouse (MGHD), conventional greenhouse (CGHD), and open sun (OSD) dryers. The drying behavior, kinetics, and quality were evaluated. The MGHD was constructed by modifying a conventional greenhouse with a fleece of black polyester material. Evaluation of air properties of the dryers without and with cocoa beans showed that the MGHD had average temperatures of 2 and 8°C above, and relative humidity of 12.28% and 25.48% below the CGHD and OSD, respectively. The drying data were fitted to four thin layer mathematical models. The Page and Overhult models gave favorable ranges of *R* ^2^ (.976 to .987), chi‐square (3.7 × 10^−4^ to 9.9 × 10^−4^), and root mean square (RMSE; 0.0188 to 0.0307) for the three dryers. The cocoa beans dried in the MGHD took a shorter time to reach the expected 5%–8% moisture content and were of grade one quality.

## Graphical abstract

Cocoa bean drying is a key quality determining process in the cocoa chain. Its drying kinetics using a modified greenhouse dryer has demonstrated a significant reduction in drying time giving high‐quality cocoa beans that meets internationally recommended standards.

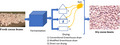

## 1 INTRODUCTION

In Cameroon, cocoa (*Theobroma cacao* L.) is a major cash crop constituting over 90% of the income earnings to growers, with a projected annual production of about 600,000 tons by 2020 (NCCB, 2017). It is the fourth world producer of cocoa, after Ivory Coast, Ghana, and Nigeria (Armathé, Mesmin, Unusa, & Soleil, 2013; Żyżelewicz et al., 2019). Largely due to drying difficulties, annual postharvest losses are estimated at 30%–40% (Ngalame, 2010). The farm(er)‐based fermentation and drying processes are not standardized and often lead to production of low‐quality beans, attracting lower prices.

After fermentation, the beans are dried to a moisture content of 5% to 8% before packaging, storage, sale, or transportation. This prevents mold infestation and allows the continuation of some chemical changes which occurred during fermentation and improve flavor development (Akhaze, 2012). A very rapid drying rate results in excessively acidic beans with case hardening (shriveling), and if drying takes longer than 7 days, mold contamination may occur. Thus, the drying rate is very critical for the final quality of cocoa beans (Bray, 2012; Kongor et al., 2016).

Open sun drying (though most popular) now seems obsolete, because it is weather‐dependent and labor‐intensive, and the food is exposed to vermin, rain, and dirt (Bala & Janjai, 2013; Sidrah, Manzoor, & Anjum, 2016). Greenhouse drying is environmentally friendly (Manoj, 2013), as high prices of fossil fuels and shortage of wood have increased the emphasis on using alternative renewable energy sources (Mühlbauer, 1986). Preferred solar dryers (including greenhouse dryers) should reduce contamination, dry faster and uniformly, giving a better quality product than open‐air methods (Nidhi, 2015; Puello‐Mendez et al., 2017).

Although greenhouse and other artificial dryers have been used globally to dry cocoa beans and other food produce (Janjai, 2012; Manoj, 2013; Nidhi, 2016), methods used in Cameroon include the open sun on cemented floors, raised wooden mats, tarred roadsides, and firewood ovens (Dopgima et al., 2015; Niemenak, Kelechi, & Chijioke, 2014). In poorly constructed or broken ovens, smoke may reach the beans leading to the production of poor colored and smoky beans with probability of developing polycyclic aromatic hydrocarbons (PAHs) considered cancerous (Ngalame, 2010). Over 2,000 tons of Cameroon cocoa was rejected in 2012 by the European Union due to smoke contamination that resulted from use of cracked firewood ovens (EURACTIV, 2013).

Simulation, construction, cost, and usage of conventional greenhouse dryers have been experimentally analyzed and described as technically and economically feasible for rural farmers in Colombia (Puello‐Mendez et al., 2017). During greenhouse drying, the product placed on trays receives solar radiation through the plastic cover and moisture is removed by natural or forced convection modes. One innovation in agricultural greenhouses is the use of a fleece material which generates higher temperatures to treat the soil (soil solarization) against nematodes and spores before planting (Clyde, Stapleton, Carl, & Devay, 1997; Stapleton, 2000). Although mathematical modeling of the drying process of cocoa beans and other produce using conventional greenhouse dryers has been documented by several authors (Nidhi, 2015; Puello‐Mendez et al., 2017), such information using a greenhouse dryer equipped with fleece is limited in literature.

In this study, the higher temperatures generated by using a fleece were explored for the drying of cocoa beans. A comparative evaluation of the performances and drying behavior of cocoa beans was done using a greenhouse dryer equipped with a polyester fleece, a conventional greenhouse dryer, and the open sun. The drying air properties (temperature and relative humidity), the drying kinetics, and quality of cocoa beans were evaluated. Mathematical modeling was done using the Page (Karathanos & Belessiotis, 1999), Henderson and Pabis (Akpinar, Bicer, & Yildiz, 2003), Lewis (Ndukwu, Ogunlowo, & Olukunle, 2010), and Overhult (Fernando & Amarasinghe, 2016) equations.

## 2 MATERIALS AND METHODS

### 2.1 Construction of the dryers

**FIGURE 1:**
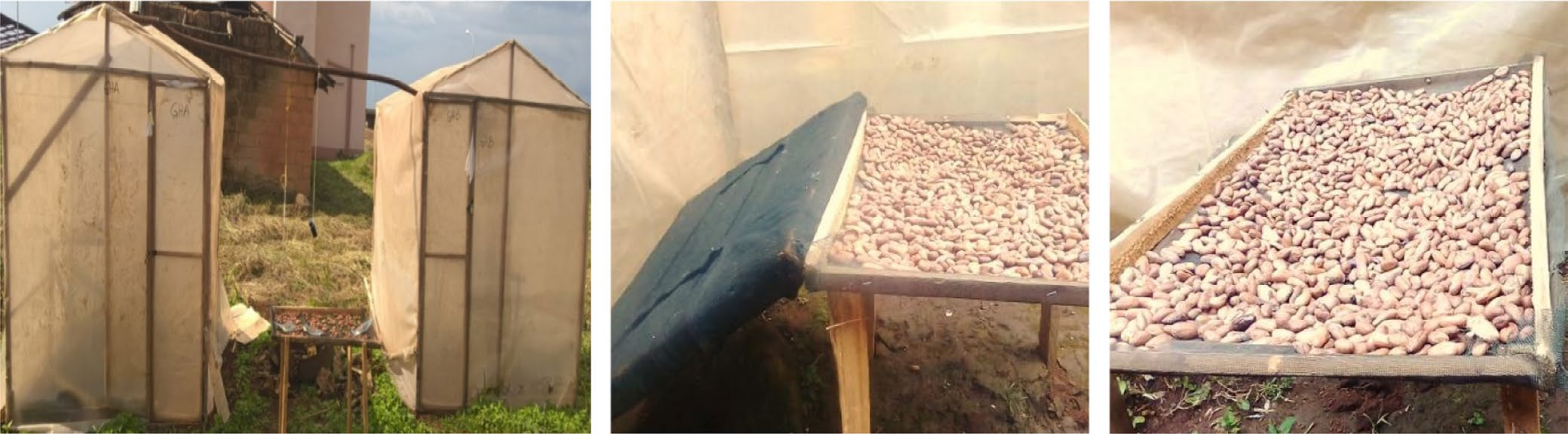
Prototype greenhouse dryers (left), internal view of MGHD (modified greenhouse) showing position of the fleece (middle), and CGHD (conventional greenhouse) (right)

This study was carried out in the campus of the University of Bamenda—Cameroon (5°59′0″N, 10°15′0″E) in November 2017. Two prototype roofed greenhouse dryers of dimension 1.5 × 1.5 × 2 m were constructed with translucent polyethylene material as described by Prakash and Kumar (2014). The modified greenhouse dryer (MGHD; Figure 1, center) was equipped with a 1‐mm‐thick black polyester fleece (specific heat capacity 1.87 J/g °C) slanted at an angle of 16° above the basal ventilator to maximize reception of solar radiation as described by Olatunbosun (2011) and Nidhi (2016). Its floor was lined with 5‐cm‐thick coarse black stone gravel for heat conservation and drying during nonsunny conditions (Reddy, 2015).

### 2.2 Evaluation of air properties of the dryers without load

Three data loggers, (Tinytag Plus 2‐TGP‐4017, Gemini Data Loggers, UK) −40 to +85°C with built‐in sensors were used. These were set to record temperature and Rh at 30‐min interval from 8:00 a.m. to 5:00 p.m. for three consecutive days. Each was hung centrally in the dryers, and data were downloaded at the end of each day.

### 2.3 Sample preparation

Ripe, fresh cocoa (*Forastero* variety) pods were obtained from a cocoa farmer in Ngie (5°59′N, 9°50′E), a locality in Bamenda, and transported to the laboratory same day of harvest. After checking for ripeness and signs of diseases, those in optimal quality were broken and beans spread on a wooden bench in the open sun (28°C and 40% RH) for 2 hr, to alter the moisture content, reduce fermentable sugars, and ensure less acid production during fermentation (Kongor et al., 2016). The basket method, with periodic opening and turning, was used for fermentation for 6 days inside the conventional greenhouse (CGHD) as described by Bray (2012) and modified by Kongor et al. (2016).

### 2.4 The drying process

The fermented beans were divided into three equal portions and dried in the three dryers. For each, three microtrays of plastic mesh (12 × 20 × 3 cm) were used into which samples of about 50 g were put. These were labeled as M_1_, M_2_, and M_3_ for MGHD; C_1_, C_2_, and C_3_ for CGHD; and S_1_, S_2_, and S_3_ for OSD. The beans were spread one layer thick on each tray. Drying started from 9 a.m. and ended at 5 p.m. daily, until the weight of the sample became constant. For the first day, the weights of the microtrays and drying temperatures were taken at intervals of 20 min for the first hour, 30 min for the second, and hourly till 5 p.m. and for the rest of the days. The beans dried in MGHD and CGHD were allowed on their respective trays throughout the nights, while that for OSD were put in khaki colored, craft paper envelope during rain and night, to mimic farm conditions where beans being dried are put in jute bags and kept in the house every evening and taken out the following morning (Bray, 2012).

### 2.5 Modeling of the drying kinetics

The linearized forms of Page, Henderson and Pabis, Lewis and Overhult equations (Table 1) were used. The moisture ratio was defined by the equation:

(1) $$M_{R}=\frac{M_{i}-M}{M_{i}-X_{f}}$$

where *M_R_* is the moisture ratio and *M*, the moisture content at time *t.*

**TABLE 1:** Equations tested for modeling drying kinetics

Name of model	Model	Linearized form of model	Graph plotted
Page	*M_R_* = exp(−*kt^n^*)	Ln(ln*M_R_*) = *n*ln*t* − ln*k*	ln(ln*M_R_*) against ln*t*
Henderson & Pabis	*M_R_* = *a* exp(−*kt*)	ln*M_R_* = −*kt* + ln*a*	ln*M_R_* against *t*
Lewis	*M_R_* = exp(−*kt*)	ln*M_R_* = −*kt*	ln*M_R_* against *t*
Overhult	*M_R_* = exp((−*kt*)*^n^*)	Ln(−(ln*M_R_*)) = *n*ln*t* – *n*ln*k*	Ln(ln*M_R_*) against ln*t*

Note

*k*, drying constant; *t*, time; *a* and *n*, dimensionless coefficients.

Linear regression analysis was done using MS Excel 2010, and the *k* and *R* ^2^ (determination coefficient) values were obtained. *R* ^2^ was the primary criterion for determining the goodness of fit. The models with *R* ^2^ closest to 1 were chosen to be best fitted in modeling the drying kinetics (Ndukwu et al., 2010). These (*k* and *R* ^2^) were used to calculate the predicted and experimental moisture values, from where the chi‐square (*χ* ^2^) and the root mean square error (RMSE) were calculated using Equations (2 and 3); Sobukola, Dairo, & Odunewu, 2008; Ndukwu et al., 2010).

(2) $$\chi^{2}=\sum_{i=1}^{n}{\left(\frac{MR_{\exp,i}-MR_{\text{pre},i}}{N-n}\right)}^{2}$$

(3) $$RMSE={\left[\frac{1}{N}\sum_{i=1}^{n}{\left(MR_{exp,i}-MR_{\text{pre},i}\right)}^{2}\right]}^{\text{1}\;/\text{2}}$$

where *MR*_exp_ *= *experimental moisture ratio, *MR*_pre_ = predicted moisture ratio, *N* = number of experimental data points, and *n* = number of constants in the model.

### 2.6 Determination of moisture content

Dry weight moisture contents (*M_i_*) of cocoa sample were taken fresh, after fermentation, and at the end of drying using the oven method described by Ismail and Idriss (2013) and Prasanna and Shruthi (2017). *M_i_* was then obtained from Equation (4).

(4) $$M_{i}=\frac{w_{2}-w_{3}}{w_{2}-w_{1}}\times 100$$

where *M_i_* = initial moisture content (g); *W*_1_ = weight of empty beaker (g); *W*_2_ = weight of moist sample + beaker (g); *W*_3_ = weight of dried sample + beaker (g).

The overall drying rate per dryer, the ratio between the differences in moisture content at the end of the drying period, was calculated according to Sekar, Sekar, & Valarmathi (2018), as follows:

(5) $$D\dot{r}=\left(\frac{M_{i}-M_{f}}{T\Delta }\right)$$

where $D\dot{r}$ = drying rate (g/h); TΔ = total hours of drying; *M_f_* = final moisture content (g).

### 2.7 Determination of pH

The pH was determined for fresh, fermented, and dried beans according to Hii, Law, and Cloke (2008), Tagro et al. (2010), and Niemenak et al. (2014) as follows: Six beans from each treatment were randomly selected, deshelled manually, and nibs ground using an electric blender (Vitamix 65542, Amazon) to give a powdered sample. To 6 g in a test tube, 20 ml of boiling distil water was added and homogenized immediately by vortexing in a high‐speed vortex mixer (XH‐D, Scientific instruments) for 2 min. The contents were filtered using a plastic mesh sieve and then through a Whatman filter paper No 1 (Camlab) and allowed to cool to room temperature (25°C). The pH of the filtrate was determined using a digital pH meter (PHS‐25, CNW & J Instruments Co. Ltd). These were done in triplicates for each sample.

### 2.8 Determination of bean color (the cut test method)

The cut test for dried beans was carried out as described by Niemenak et al. (2014). From each batch of cocoa beans dried in MGHD, CGHD, and OSD, 100 beans were taken out randomly and cut lengthwise using a sharp surgical blade. The cut beans were placed facing upwards on a white background, examined with the naked eyes in full daylight, and snapped using a 16 megapixels high‐resolution digital SLR camera (D420, Nikon). They were observed for pale brown, dark brown, slaty, violet, violet brown, moldy, and moldy‐infested and expressed as a percentage of the total beans. These were compared to standards set by Niemenak et al. (2014), considering that the best bean quality in terms of color decreases from pale brown to brown, while the slaty, violet, violet‐brown, and moldy to infested are considered to be of poor quality (Amoah‐Awua, Schwan, & Fleet, 2014).

## 3 RESULTS AND DISCUSSION

### 3.1 Air properties of dryers under no load and load conditions

Temperature and relative humidity (RH) changes were observed to have a direct influence on the drying rate of cocoa beans.

#### 3.1.1 No load condition

**FIGURE 2:**
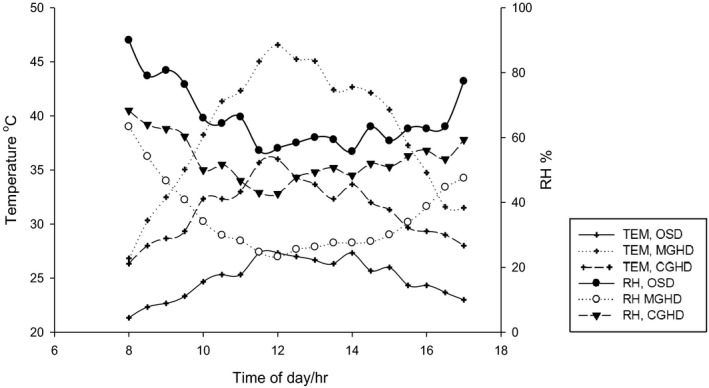
Temperature (TEM) and relative humidity (RH) profiles of MGHD, CGHD, and OSD (open sun) dryers under no load

The comparative averages of daily temperature and relative humidity profiles for three consecutive days in the three dryers are shown in Figure 2. In all the dryers, temperature increased from low values at 9 a.m. to maximum around noon and then decreased steadily to 5 p.m. as expected under natural conditions. Relative humidity decreased from high values at 9 a.m. to minimum around noon and then increased in the later parts of the day for all the dryers. The highest relative humidity is exhibited in OSD and least in MGHD, while the highest temperature was observed in the MGHD and least in OSD. The differences in temperature and relative humidity between MGHD and OSD, and CGHD and OSD at 9:00 a.m., 12 noon, and 5:00 p.m. are shown in Table 2.

**TABLE 2:** Temperature and RH changes under no load condition

	MGHD	Temperature (°C) CGHD	OSD	Δ TEM	
				MGHD & OSD	CGHD & OSD
9:00 a.m.	35.05 ± 6.82	29.33 ± 3.21	23.33 ± 1.53	11.72 ± 5.29	6 ± 1.68
12 noon	46.56 ± 3.29	36 ± 2.00	27.33 ± 2.08	19.23 ± 1.21	8.67 ± 0.08
5:00 p.m.	31.51 ± 4.53	28 ± 5.00	23 ± 1.73	8.51 ± 2.80	5 ± 2.20

	RH (%)				Δ RH
9:00 a.m.	40.8 ± 15.15	60.33 ± 16.50	76.33 ± 19.14	−35.53 ± 3.99	−16 ± 2.64
12 noon	23.23 ± 4.70	42.67 ± 5.03	56.67 ± 12.22	−33.44 ± 7.52	−14 ± 7.19
5:00 p.m.	47.47 ± 11.48	59.33 ± 19.14	77.33 ± 13.01	−29.86 ± 1.53	−18 ± 6.13

The fairly higher temperature and lower RH differences between MGHD and OSD than between CGHD and OSD show that MGHD has higher drying air potentials than CGHD throughout the day (Figure 2). The MGHD and CGHD are shielded from wind and rain, thus, together with the greenhouse effect are likely responsible for the higher temperatures and lower RH recorded. The ΔTEM and ΔRH values between MGHD and the two differ, indicating that the fleece in MGHD has a positive influence on its drying air properties. This is in line with Prakash and Kumar (2014), and Nidhi (2016) who observed average temperature differences between CGHD and OSD of 6–8°C throughout the day, thus confirming the significant influence of the fleece in the heating of MGHD.

#### 3.1.2 Load condition

**FIGURE 3:**
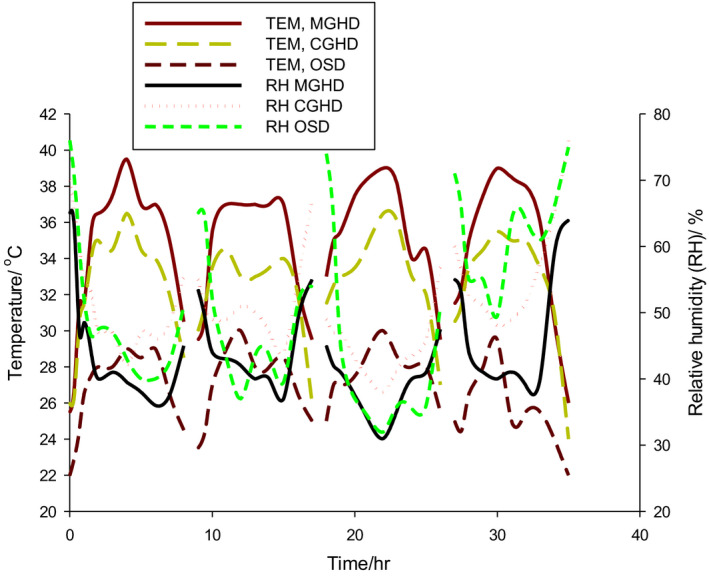
Temperature (TEM) and relative humidity (RH) profiles of drying air in the loaded dryers

Figure 3 shows the comparative averages of daily temperature and RH profiles during the drying process. The variations showed similar trends as observed for no load conditions. The highest average TEM/RH was 34.37°C/44.01%, 32.29°C/52.21%, and 26.54°C/52.21% for MGHD, CGHD, and OSD, respectively. Temperature differences at 9:00 a.m., 12 noon, and 5:00 p.m. between MGHD and OSD and CGHD and OSD varied from 4.7°C to 8.7°C and 2.2°C to 5.2°C, respectively. The corresponding variations in RH were −14.83% to −0.5% and −11.83% to −6.67% indicating that MGHD had higher drying potentials than the CGHD and OSD. This was further supported by the observation that the overall average temperature differences between OSD and MGHD were 7.83°C and 5.75°C between OSD and CGHD.

Comparing these results with those under no load condition (Figure 2) shows a reduction in temperature and an increase in the corresponding RH in the three dryers. This could be attributed to the fact that during drying, heat is used to convert moisture in the product to vapor which is then released to the dryer thus raising the relative humidity. The highest average temperatures recorded were 39.5°C, 36.5°C, and 29.5°C for MGHD, CGHD, and OSD, respectively, between the 12th and 14th hour of the day with least RH recorded within the same time except for rainy periods. Throughout the drying period, MGHD exhibited the least RH and highest temperature, and these favored the drying of cocoa beans over CGHD and OSD. Since the MGHD and CGHD were the same in all aspects except the presence of the fleece in MGHD, the increase in the temperature and reduction in RH in MGHD over CGHD were directly attributed to the influence of the fleece that was absent in CGHD.

### 3.2 Drying kinetics

**FIGURE 4:**
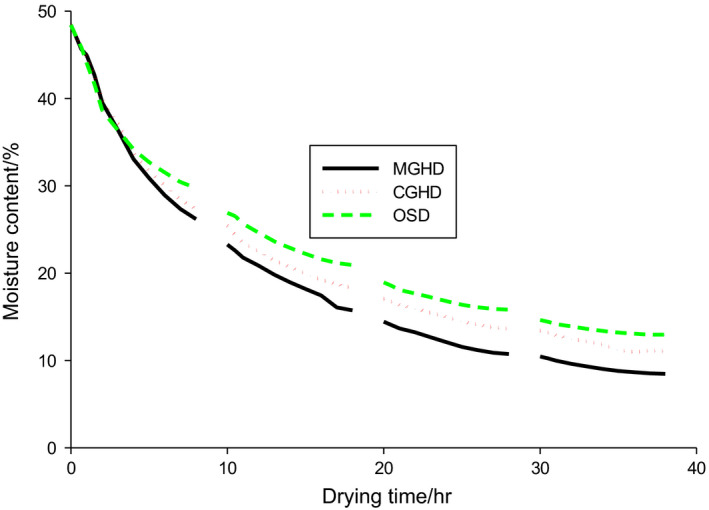
Drying curves for cocoa beans in MGHD, CGHD, and OSD. The breaks in the curves represent the night periods of no drying

The drying curves for the three dryers are shown in Figure 4. The rate of moisture loss is higher at the beginning (when the moisture content of the beans is high) and reduces wit time, leading to a reduction in bean weight. The testa hardens and becomes brittle, while the cotyledons shrink leading to a reduction in length, thickness, and breadth of the bean. The moisture content decreased continuously with drying time and attained stable values after the third day. The rate of moisture loss from the second day was higher for MGHD than CGHD and OSD, respectively, thus confirming the positive impact of the fleece in the drying process.

The moisture content reduced from 48.42% to 5.95, 9.06, and 9.78% in MGHD, CGHD, and OSD, respectively, during the 4 days of drying. Moisture contents between 6% and 8% are considered good for storage. During the three nights, moisture loss ranged from 0.47 ± 0.04% to 6 ± 0.38% (as a percentage of the initial moisture content) in both dryers. Beans from OSD stored in brown khaki envelopes overnight showed the highest percentage moisture loss during the nights, followed by the beans in MGHD (Table 3). This could be due to the residual heat in the beans when the heated air/sunlight is no longer available (Ndukwu et al., 2010). These losses account for the breaks observed in the drying curves. These results were similar to those of Hii et al. (2008), whose night losses ranged from 1% to 5% per night. There was decrease in the amounts of moisture lost from the second and third nights compared to that lost in the first night and could be attributed to the decrease in the overall moisture contents of the cocoa beans as the drying proceeded.

**TABLE 3:** Regression analysis for drying rate curves

	a	*n*	*R* ^2^
MGHD	1.295	1.001	.8511
CGHD	1.212	0.981	.8211
OSD	1.263	0.942	.8188

Although cocoa has been demonstrated to exhibit a constant rate drying period at above 70% moisture content (dry basis) as do most agricultural produce (Ndukwu et al., 2010), there was no constant rate drying period observed in this work. This was attributed to the fact that the initial moisture content was less than 70%. The free bound water that would have been lost during the constant drying rate phase was probably lost during fermentation as shown by a significant decrease in the moisture content of the fermented beans from the fresh one of 50.68 ± 00 to 48.42 ± 0.72%. For the first falling rate period observed in day 1, the movement of moisture within the beans is likely governed by diffusion and capillarity since they are not saturated with water, while that for the second falling rate period from day 2 can be attributed to flow due to shrinkage, pressure gradients, and gravity (Akhaze, 2012; Hii et al., 2008). The higher drying rate in MGHD could equally be attributed to its lower relative humidity which favored the carrying away of the evaporated moisture from the surfaces of the cocoa beans (Ndukwu et al., 2010).

#### 3.2.1 Drying rate curves

**FIGURE 5:**
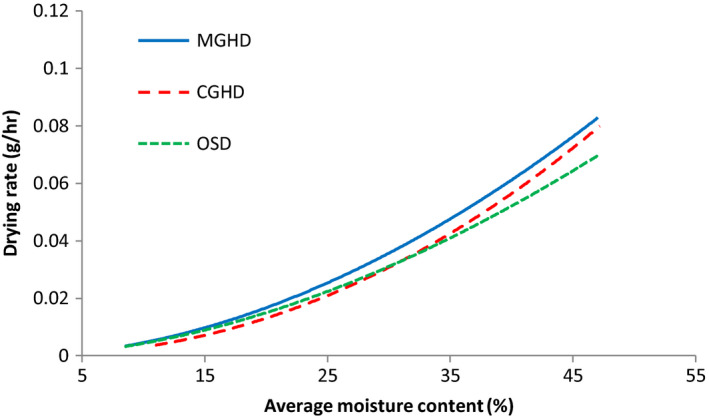
The drying rate curves for cocoa beans in MGHD, CGHD, and OSD. The rates are calculated as a function of moisture loss per hour

The drying rate (Figure 5) showed only the falling rate period (as observed for most agricultural products), indicating the loss of free moisture during predrying treatment (fermentation). Drying rates decreased in a linear manner with moisture content and increased in drying time. They varied from 0.07–0.00089, 0.06–0.001, and 0.07–0.0018 g/hr for MGHD, CGHD, and OSD, respectively. The variations were not regular because drying was dependent on fluctuating weather conditions. These could be described by an equation of the form:

(6) $$\frac{\mathrm{dX}}{\mathrm{dt}}=aX^{n}$$

where $\frac{\mathrm{dX}}{\mathrm{dt}}$ is the drying rate at time *t*, *X* is the moisture content, and *a* and *n* are constants. The observed *R* ^2^ values range from .818 to .851(Table 3).

Using Equation (5), overall drying rates obtained were 1.21, 1.13, and 1.01 g/hr, giving the estimated time to dry cocoa beans to the first‐grade moisture content of 5%–6% to be 4, 5, and 6 days in the MGHD, CGHD, and OSD, respectively. These estimates for CGHD and OSD are in line with those of Puello‐Mendez et al. (2017), Prasanna and Shruthi (2017), and Sekar et al. (2018) who observed that 4 and 6 days were required to dry cocoa beans to moisture content of 7% using the CGHD and OSD, respectively. This reduction in drying time using the MGHD clearly indicated the positive influence of the fleece in improving the drying air conditions and consequently the drying rate of cocoa beans.

#### 3.2.2 Modeling of the drying kinetics

**FIGURE 6:**
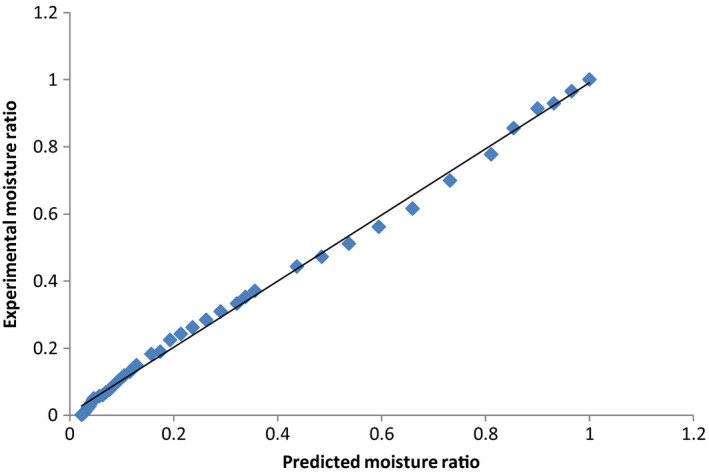
Comparison of experimental with predicted moisture ratios for MGHD using the Page and Overhult models

**FIGURE 7:**
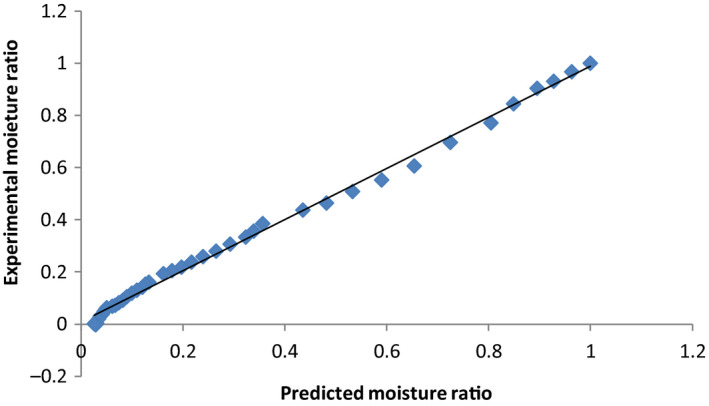
Comparison of experimental with predicted moisture ratios for CGHD using the Page and Overhult models

**FIGURE 8:**
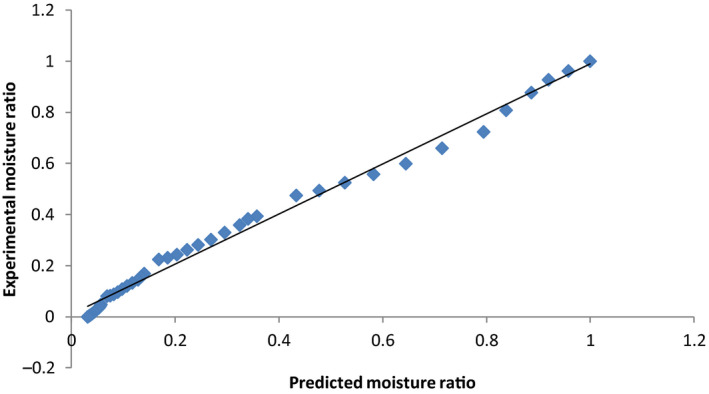
Comparison of experimental with predicted moisture ratios for OSD using the Page and Overhult models

Data on moisture content were converted to moisture ratio (Equation 1), and the curve fitting procedure was performed for linearized forms of Lewis, Handerson and Pabis, Overhult and Page models. From the equations of the line, the drying constants, *k*, *a*, *n*, and *R* ^2^ values (Table 4) and regression analysis were obtained. From these, the experimental and predicted values for the Page and Overhult models that were best fitted and had highest *R* ^2^ values were calculated. The plot of experimental versus predicted moisture ratios equally gave very high *R* ^2^ (Figures 6, 7, 8) and lower chi‐square and RMSE values (Table 4). These results are in line with the observations of Sobukola et al. (2008) and Ndukwu et al. (2010) who reported that the Lewis, Henderson, and Parbis models were good in modeling the drying kinetics of cocoa under isothermal conditions.

**TABLE 4:** Calculated *k*, *n*, *R* ^2^, chi‐square, and RMSE values for the Page and Overhult models

	Page			Overhult		
	MGHD	CGHD	OSD	MGHD	CGHD	OSD
k	0.002	0.002	0.003	0.002	0.002	0.002
*n*	1.011	0.981	0.942	1.011	0.981	0.942
*R* ^2^	.987	.985	.976	.987	.985	.976
*χ* ^2^	0.00037	0.00045	0.00099	0.00037	0.00045	0.00099
RMSE	0.0188	0.0207	0.0307	0.0188	0.0207	0.0307

### 3.3 Quality of dried cocoa beans

#### 3.3.1 Moisture content

Table 5 shows the bone‐dry moisture contents of fresh, fermented, and dried cocoa beans. Final moisture content of beans from MGHD was significantly lower than that of the CGHD and OSD. According to Peláez, Saulo, and David (2016), the reduction in moisture content during fermentation could be attributed to the fact that fermentation of cocoa bean pulp by microbial action caused cell rupture and release of intracellular juices, thereby reducing the amount of moisture retained by beans. He equally observed a reduction in moisture content from 51.89 ± 1.74% (fresh cocoa beans) to 47.07 ± 0.60% after 144 hr of fermentation which was within the degree of reduction (50.68%–48.42%) observed in this study. These results showed that the cocoa beans dried in the MGHD were of grade one quality (CAOBISCO/ECA/FCC, 2015), while that dried in CGHD and OSD still needed more time to dry.

**TABLE 5:** Moisture content and pH of fresh, fermented, and dried Cocoa beans

	Moisture content (%)			pH		
	MGHD	CGHD	OSD	MGHD	CGHD	OSD
Fresh	50. 7 ± 0.2	50.7 ± 0.2	50. 7 ± 0.2	5.8 ± 0.2	5.8 ± 0.2	5.8 ± 0.2
Fermented	48.4 ± 0.7	48.4 ± 0.7	48.4 ± 0.7	4.97 ± 0.05	4.97 ± 0.05	4.97 ± 0.05
Dried	5.95 ± 1.29	9.06 ± 0.49	9.78 ± 1.08	4.65 ± 0.40	4.8 ± 0.46	5.34 ± 0.36

#### 3.3.2 pH

Table 5 shows average pH values obtained for the fresh, fermented, and dried cocoa beans in the three dryers. That of the fermented cocoa beans (4.97) falls within the recommended range of 4.7–5.2 as stated by Afoakwa, Kongor, Budu, Mensah‐Brown, and Takrama (2015) and Peláez et al. (2016). Poorly fermented cocoa beans have a pH range of 5.5–5.8. This shows that the cocoa beans used in this research were properly fermented. During drying, pH decreased from 4.97 to 4.65 and 4.80 in the MGHD and CGHD, respectively, while that of OSD increased to 5.34. These pH variations could be attributed to rapid drying and case hardening that prevented outwards migration of excess acetic acid in beans (Guehi et al., 2010; Hii, Abdul, Jinap, & Che Man,^2006^), and differential drying rates observed in the three dryers. Nonenzymatic reactions, to form volatile fractions like pyrazines, might have equally occurred leading to oxidization and polymerization of polyphenols as observed by Lærke (2010) and Kongor et al. (2016). The pH of cocoa beans in MGHD could have been higher if the beans were dried in thicker layers according to Hii et al. (2008), who observed a pH increase from 4.91 to 5.39 with increase in loadings. CAOBISCO/ECA/FCC (2015) describes dried cocoa beans with pH of ≤5 as acidic and recommends that for pH to be increased in any drying method, cocoa beans should be dried in layers ≥5 cm thick and turned regularly.

#### 3.3.3 Bean color

Table 6 shows the cut test results of the dried cocoa beans. The insignificant slaty cocoa beans observed probably resulted from fermentation lapses and not the drying process (Afoakwa, 2014; Niemenak et al., 2014). Grade one quality cocoa beans should contain ≤3% slaty, moldy, and infested beans (Barbara et al., 2015; CAOBISCO/ECA/FCC, 2015; Ngalame, 2010). This shows that the dried cocoa beans in terms of color are of good quality.

**TABLE 6:** Color observations for the dried cocoa beans as percentage of cut beans

Drying media	No of cut beans	Dark brown	Pale brown	Slaty	Violet brown	Violet	Purple	Moldy	Moldy and infested
MGHD	100	11	88	1	—	—	—	—	—
CGHD	100	14	85	1	—	—	—	—	—
OSD	100	5	95	—	—	—	—	—	—

## 4 CONCLUSION

The fleece has a significant influence on increasing temperature and reducing drying time by 20% and 33.3% in the MGHD compared to CGHD and open sun. The Page and Overhult models are best fitted for modeling the drying kinetics of cocoa beans in the three dryers. The quality of the cocoa beans dried in MGHD in terms of moisture content and bean color is of first grade compared to that dried in the CGHD and OSD. If further studies could be done in simulating this dryer, varying the material, thickness, and orientation of the fleece, it could give better drying properties for the drying of cocoa beans and other agricultural produce.

## CONFLICT OF INTEREST

The authors declare they do not have any conflict of interests.

## **AUTHOR** **CONTRIBUTION**

Frederick D. Banboye conceived and designed the experiments, performed the experiments, analyzed and interpreted the data, contributed reagents, materials, analysis tools, or data, and wrote the paper. Martin N. Ngwabie contributed reagents, materials, analysis tools, or data and wrote the paper. Silvia A. Eneighe performed the experiments, contributed reagents, materials, analysis tools, or data, and wrote the paper. Divine B. Nde conceived and designed the experiments, analyzed and interpreted the data, contributed reagents, materials, analysis tools, or data, and wrote the paper.

## ETHICAL APPROVAL

This study does not involve any human or animal testing.
